# Raman Microscopic Analysis of Dry-Surface Biofilms on Clinically Relevant Materials

**DOI:** 10.3390/microorganisms10071369

**Published:** 2022-07-07

**Authors:** Thomas J. Tewes, Isabella Centeleghe, Jean-Yves Maillard, Frank Platte, Dirk P. Bockmühl

**Affiliations:** 1 Faculty of Life Sciences, Rhine-Waal University of Applied Sciences, Marie-Curie-Straße 1, 47533 Kleve, Germany; thomasjohann.tewes@hsrw.eu (T.J.T.); frank.platte@hsrw.eu (F.P.); 2 School of Pharmacy and Pharmaceutical Sciences, Cardiff University, Cardiff CF10 3NB, Wales, UK; centeleghei@cardiff.ac.uk (I.C.); maillardj@cardiff.ac.uk (J.-Y.M.)

**Keywords:** dry-surface biofilms, Raman microspectroscopy, *Staphylococcus aureus*, *Bacillus licheniformis*, support vector machine, stainless steel, PVC

## Abstract

Moist/hydrated biofilms have been well-studied in the medical area, and their association with infections is widely recognized. In contrast, dry-surface biofilms (DSBs) on environmental surfaces in healthcare settings have received less attention. DSBs have been shown to be widespread on commonly used items in hospitals and to harbor bacterial pathogens that are known to cause healthcare-acquired infections (HAI). DSBs cannot be detected by routine surface swabbing or contact plates, and studies have shown DSBs to be less susceptible to cleaning/disinfection products. As DSBs are increasingly reported in the medical field, and there is a likelihood they also occur in food production and manufacturing areas, there is a growing demand for the rapid in situ detection of DSBs and the identification of pathogens within DSBs. Raman microspectroscopy allows users to obtain spatially resolved information about the chemical composition of biofilms, and to identify microbial species. In this study, we investigated *Staphylococcus aureus* mono-species DSB on polyvinylchloride blanks and stainless steel coupons, and dual-species (*S. aureus*/*Bacillus licheniformis*) DSB on steel coupons. We demonstrated that Raman microspectroscopy is not only suitable for identifying specific species, but it also enables the differentiation of vegetative cells from their sporulated form. Our findings provide the first step towards the rapid identification and characterization of the distribution and composition of DSBs on different surface areas.

## 1. Introduction

In the natural environment, bacteria reside on surfaces as biofilms, aiding in their protection from external stressors such as UV, antimicrobial agents, and dehydration [1]. Environmental surfaces, including high-touch surfaces such as commodes, bed rails, and keyboards, provide an ideal substrate for biofilm formation [2]. Limited moisture, nutrient sources, and routine disinfection and cleaning protocols provide conditions for the formation of dry-surface biofilms (DSBs) in healthcare environments [3]. There are a number of studies that provide evidence supporting the widespread presence of DSBs on healthcare surfaces worldwide [3,4,5]. In addition, DSBs are more resistant to chemical and physical treatments than hydrated biofilms, including exposure to 20,000 ppm hypochlorite [6] or heat treatment [7], and mechanical removal seems essential in the efficacy of products against DSBs [8].

The use of Raman microspectroscopy to differentiate microorganisms is not only feasible but can be also very precise [9,10,11,12]. Raman spectra of microorganisms from pure cultures grown under standardized conditions are specific enough to differentiate individual species when using appropriate data and mathematical methods. However, environmental biofilms must be assumed to consist of diverse microorganisms in various metabolic states but also contain extracellular polymeric substances (EPSs) [13], which alone produce strong Raman intrinsic signatures [14,15], and might interfere with or completely mask the microbial Raman signals. Depending on the excitation wavelength, fluorescence can also lead to the masking of the signals. Thus, the identification of microorganisms within a biofilm might strongly be affected by the biofilm matrix. Consequently, a mathematical model to detect individual species within biofilms would need to have reference data adjusted to all relevant environments, or computationally circumvent disturbing influences. As an exception to this assumption, species with very specific Raman bands and strong signals, such as those caused by colored pigments such as carotenoids, might be detected more easily.

Ivleva et al. demonstrated the feasibility of Raman microscopy for the study of multispecies hydrated biofilms in 2009 [15]. In this study, corresponding characteristic regions were identified for different biofilm components within the spectra, and detailed chemical information about these matrix components could be provided, without, however, focusing on the characterization of the microbial species in the biofilm themselves. In 2012, Beier et al. studied two-species bacterial hydrated biofilms with microbial species of importance for dental health: *Streptococcus sanguinis* and *Streptococcus mutans* [16]. They developed an algorithm based on data from hydrated biofilms dried on surfaces and made predictions about species in hydrated biofilms [16].

Here, we explore the extent to which Raman microspectroscopy is suitable (i) to detect DSBs on two different surfaces: polyvinylchloride (PVC) and stainless steel surfaces, and (ii) to detect individual species and (iii) the quantitative distribution of the bacterial species within DSB.

## 2. Materials and Methods

### 2.1. Bacterial Growth

*Staphylococcus aureus* NCTC 10788 and spore-former *Bacillus licheniformis* ATCC 14580 were used to form the DSB. These bacterial species are commonly found in environmental DSBs [5]. Overnight cultures of each species were prepared in 20 mL of sterile tryptone soya broth (TSB; Oxoid Ltd., Hampshire, UK) and grown aerobically at 37 °C with shaking at 120 rpm. Cultures were centrifuged at 5000× *g* for 10 min the following day and washed with TSB. The bacterial suspensions were adjusted to 10^6^ CFU/mL.

Bacterial enumeration was performed by plating 3 × 0.01 mL drops of bacterial suspension on tryptone soya agar (TSA; Oxoid, Thermo Fisher Scientific, Waltham, MA, USA) following serial dilution in tryptone sodium chloride (1 g of tryptone (Oxoid Limited) and 8.5 g of sodium chloride (Sigma-Aldrich, Dorset, UK) in 1 L of distilled water) and incubation of TSA plates at 37 °C for 24 h [17].

### 2.2. Preparation of the Dry Surface Biofilms

All DSB were formed on disks placed in a Corning Costar flat bottom 24-well (Fisher Scientific, Loughborough, UK) cell culture plate over a 12-day period using sequential 48 h dehydration and hydration phases [17]. For monospecies DSB, each well was inoculated with TSB containing washed bacterial inoculum (10^6^ CFU/mL) and 3 g/L bovine serum albumin (BSA) (Fisher BioReagants, Fisher Scientific, Loughborough, UK) at the initial hydration phase. The well plates were incubated at room temperature (21 °C) for 48 h, with shaking at 200 rpm. All media was then drained out and the well plates containing the DSB were left to incubate at 37 °C for 48 h. This process was repeated until a mature DSB was formed at 12 days. Each hydration phase included the addition of 1 mL of 3 g/L BSA in TSB solution only. The addition of BSA enables a more consistent DSB to be formed [17]. The composition of the mature DSB following 12 days of sequential hydration/dehydration phase has been described in [17].

Monospecies DSBs of *B. licheniformis* were grown on sterile stainless-steel disks AISI 430 (0.7 ± 0.07 mm thickness; 10 ± 0.5 mm diameter) (Goodfellow Cambridge Limited, Huntington, UK). Monospecies DSBs of *S. aureus* were grown on sterile polyvinyl chloride (PVC) with a Polyurethane (PUR) coating (Goodfellow Cambridge Limited, Huntington, UK), cut into squares measuring 1 cm × 1 cm, with a thickness of 2 mm. In this paper, stainless-steel disks are referred to as coupons and the PVC pieces as blanks.

Dual-species DSB were also grown on stainless-steel disks. However, the initial bacterial inoculum consisted of 10^6^ CFU/mL *B. licheniformis* and *S. aureus* with 3 g/L BSA. Species were added in the ratio of 1:0.5, which was found to produce the most consistent dual DSB (data not shown).

### 2.3. Sample Preparation for Raman Measurements and Spectral Recording

For all Raman microscopic measurements, the same Raman system (inVia Renishaw, Gloucestershire, UK) was used. The applied helium–neon (He-Ne) laser had an excitation wavelength of 633 nm with about 7 mW on the sample at full laser power using a 100× lens with a numerical aperture of 0.85. The laser diameter was about 7.5 μm. Measurements took place in static mode with a fixed region from about 606 cm^−1^ to 1736 cm^−1^. The spectral resolution was about 1.1 cm^−1^. The default internal cosmic ray filter of the Wire 4.3 Software (Renishaw, Gloucestershire, UK) was activated during all measurements. The PVC blanks and the stainless-steel coupon were always placed on a microscopic glass slide using sterile tweezers and placed under the Raman microscope.

**PVC blanks:** Two areas of an *S. aureus* monospecies biofilm on a PVC blank were randomly selected using the 100× lens. One rectangular area with spots measuring 7 × 8 with 5 µm between every spot and another area with spots measuring 16 × 10 and a 5 µm distance between each spot were selected in the Wire 4.3 Software. The areas investigated are thus 1050 and 3375 µm^2^ in size. The laser power on the sample was about 7 mW, and the exposure time of 1 s and 10 accumulations for each spectrum was recorded.

**Stainless-steel coupons:** In less overgrown areas, clearly visible single cells within monospecies biofilms of *B. licheniformis* were targeted with the 100× lens. The cells were measured with about 3.5 mW laser power with a 2 s exposure time and 30 accumulations per spectrum. For these monospecies DSBs, in order to reduce fluorescence interference, 5 min photobleaching was performed at the same laser power. Photobleaching was not used for any other measurements. Due to the above-mentioned laser diameter, it cannot be completely excluded that Raman spectroscopic information of the near-ambient matrix was also included in the spectra. In the Supplementary Data S1, the microscopy images of some investigated single cells are shown as examples.

Random spots on the surface of the dual-species (*S. aureus/B. licheniformis*) biofilms on stainless steel coupons were located using the 100× lens. Rectangular measurement areas were selected via the Raman software Wire 4.3. The measuring distances from one point to the next were 2.5 µm. Two areas were selected on two steel coupons each, i.e., a total of four Raman mappings on stainless-steel surfaces. The examined areas are on average about 5000 µm^2^ in size. The laser power on the sample was about 3.5 mW, the exposure time was 1.5 s, and 10 accumulations for each spectrum were recorded. The acquisition time of a Raman map under these measurement parameters is approximately 3.5 h.

### 2.4. Data Preprocessing and Mapping Representation

The Raman spectra recorded from the PVC surface and the spectra recorded from single cells in monospecies biofilms were baseline corrected and smoothed via the low pass filter (LPF) and normalized (z-score). The corresponding MATLAB (R2021b, MathWorks, Natick, MA, USA) code can be found in Supplementary Data S2.

The Raman mappings from the stainless-steel surfaces were baseline subtracted with the default ″intelligent fit″ function in Wire 4.3 (polynomial order: 11, noise tolerance: 1.5, anchor end-points of intelligent polynomial: enabled) and z-score-normalized. To visualize *S. aureus*-specific signals, the intensities at point 1524 cm^−1^ were colored from black to yellow, starting at 1.5 normalized counts to the maximum value at this point on the corresponding Raman map. The slightly less specific band at 1448 cm^−1^ was colored blue according to the same principle to indicate *B. licheniformis* and the peak at 1015 cm^−1^, which is highly specific for *Bacillus* spores, was highlighted in red. Signals below the limit of 1.5 normalized intensities were made transparent to make the signal overlay visible.

### 2.5. Predictive Model for the Identification of Microorganisms

Models for the prediction of unknown microorganisms based on their Raman spectra are in principle only suitable for organisms that have been cultivated under the same conditions and are analyzed with an identical measurement setup, depending on the database. Some Raman signatures of certain microorganisms can, however, be so specific that predictive models containing only a certain type of reference spectra can also make successful predictions for Raman spectra of microorganisms recorded under different conditions. In previous research, models have been developed to distinguish 21 species from pure cultures [18]. Although these models are not designed for this in principle, one of them was applied in this work to verify to what extent predictions can be made for the microorganisms present in the DSB. This model is based on a support vector machine (SVM) with a cubic kernel function using the first 10 principal components (PCs). Before applying the model to the spectra of the dual-species DSB on stainless-steel coupons, the data were appropriately pretreated as required for the model (baseline correction and smoothing via LPF and normalization (z-score)). The exact specifications of the model can be found in [18].

## 3. Results and Discussion

### 3.1. Raman Mapping of Monospecies DSBs on a PVC Blank

**Figure 1:**
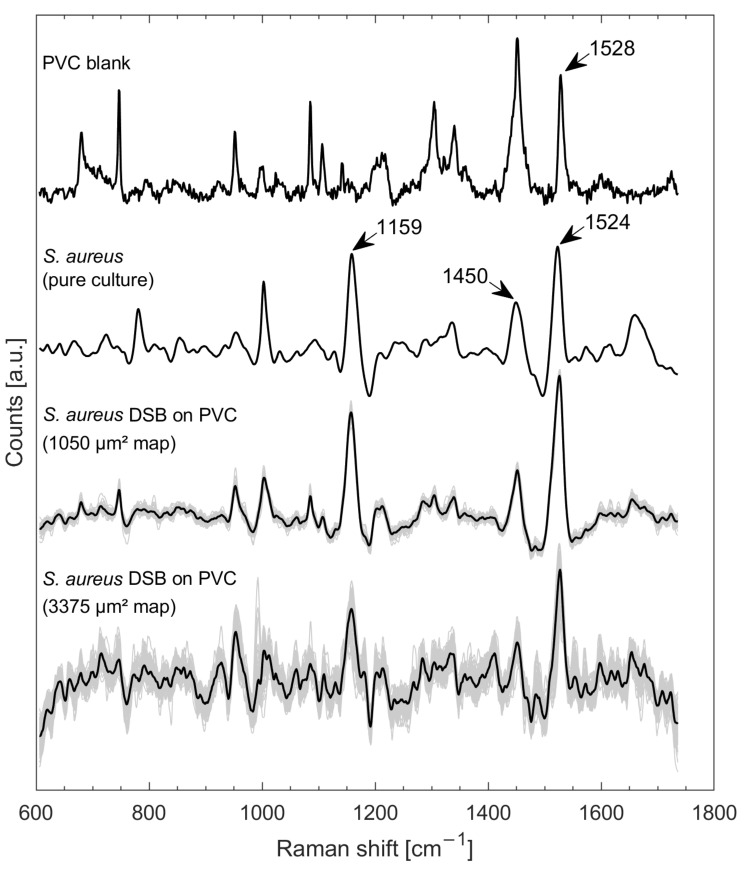
Raman spectrum from PVC blank, mean spectrum of *S. aureus* from pure culture on silver mirror slide (taken from [18]), all spectra of a rectangular Raman mapping of an *S. aureus* DSB on PVC with about 1050 µm^2^ in grey with mean spectrum in bold, and all spectra in grey along with mean spectrum in bold of a rectangular Raman mapping of another location with 3375 µm^2^ area. All spectra were baseline corrected and smoothed via LPF and normalized (z-score).

The PVC blanks showed strong Raman intrinsic signatures (Figure 1), which were caused by a blueish dye in the material (check against a Raman spectroscopic database (KnowItAll, John Wiley & Sons, Inc., Hoboken, NJ, USA)). In this regard, the PVC showed some Raman bands that occur at very similar locations in the *S. aureus* spectrum, e.g., the band triggered by the carotenoid at about 1524 cm^−1^ [19] and also the band caused by CH_2_ deformation vibrations (lipids, amino acid side chains) at about 1450 cm^−1^ [9] (Figure 1). However, it was clear that the *S. aureus* DSB spectra with 1050 µm^2^ shares high similarity to the *S. aureus* spectrum from the pure culture. In particular, the carotenoid band at about 1159 cm^−1^ [19] occurred only in the spectra of samples containing *S. aureus* and not in the PVC background. The quality of the spectra from the smaller Raman map (1050 µm^2^) was noticeably better than that of the spectra from the larger Raman map (3375 µm^2^).

Raman spectroscopic detection of species or constituents in biofilms may be limited by the substrate on which the biofilm is located, especially with very thin biofilm layers. For example, no bacteria could be detected on porcelain due to strong signals of the surface material (data not shown). *S. aureus* DSBs formed on stainless steel coupons have been shown to have an important thickness, composed of several layers of cells [17]. Although not studied on PVC, we assumed that *S. aureus* also formed several bacterial layer thicknesses (microscopic confirmation), providing a sufficient thickness to obtain strong signals (Figure 1, 1050 µm^2^ map), and making interfering background signals less relevant. If signal yield in front of an interfering background is high enough, it is possible to subtract the background spectrum to obtain meaningful data. Since the poorer signals for the greater area as shown in Figure 1 (3375 µm^2^ map) were due to stronger fluorescence interferences (recognizable from the untreated spectra (Supplementary Data S3)), it must be assumed that some of the DSB areas might carry more interfering substances (fluorophores) than others.

### 3.2. Raman Measurements of Single Cells in DSBs

**Figure 2:**
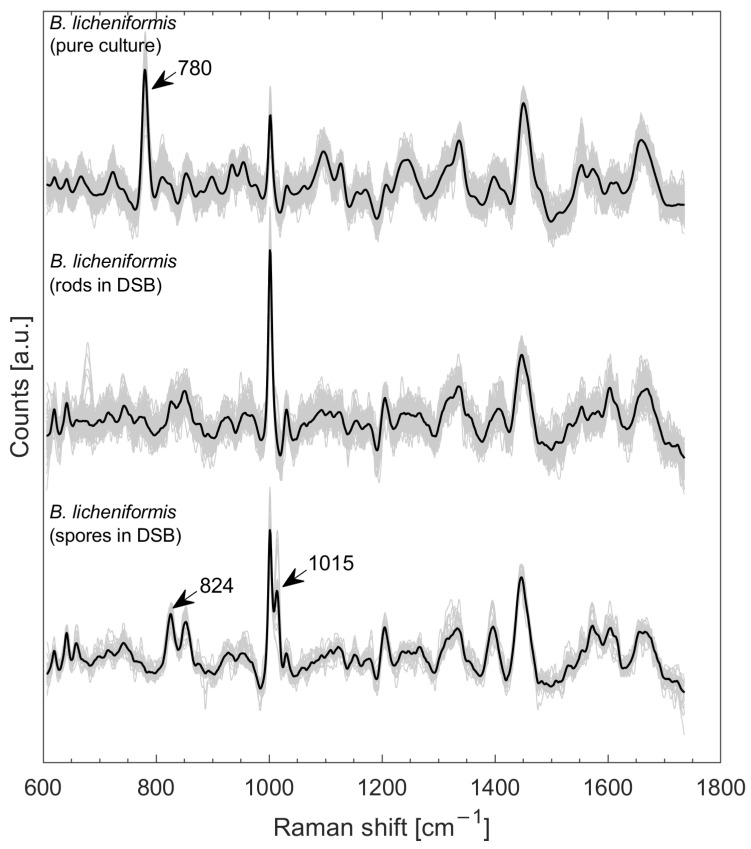
Raman spectra of *B. licheniformis* from pure culture *n* = 1240 (taken from [18]), *B. licheniformis* spectra of single stick-shaped cells from monospecies DSBs on steel coupons (*n* = 104) and spectra of spores on monospecies DSBs (*n* = 31) on steel coupons.

Figure 2 compiles the Raman spectra of *B. licheniformis* from pure culture measured on a silver mirror slide (data taken from [18]), *B. licheniformis* spectra of vegetative cells from mono species DSB on steel coupons (*n* = 104), and spectra of spores from mono species DSB (*n* = 31) on steel coupons. Exemplary microscopic images of the targeted single cells can be taken from Supplementary Data S1. The major difference between the spectra of *B. licheniformis* from pure culture and those on the monospecies DSB is represented by the strongly pronounced signature at about 780 cm^−1^ in the pure culture spectra, which is caused by a phosphate bond, cytosine, uracil, or thymine [20,21], and the much more pronounced peak at about 1003 cm^−1^ (phenylalanine [22]) of *B licheniformis* from the monospecies DSB. *Bacillus* spores were clearly recognizable by their signature at about 1015 cm^−1^, which reflects the symmetric ring ″breathing″ of dipicolinic acid (DPA) and a distinct band at 824 cm^−1^ (assigned to the vibration mode of the CH out-of-plane deformation of DPA) [23]. DPA in a complex with calcium ions (Ca-DPA) comprises up to 15% of the dry weight of *Bacillus* spores [23].

The investigations of monospecies DSB (Figure 2) showed that robust spectra can be obtained on stainless steel, even for low microbial loads, due to the favorable measurement environment for Raman spectroscopy. Being able to find valid signals, especially with little contamination, is essential for applications in situ. For example, studies have shown an important reduction in bacterial viability following disinfection and wiping [6,8], suggesting that detection of a DSB immediately post-intervention might be difficult.

### 3.3. Raman Mapping of dual-species DSBs on Steel Surfaces

**Figure 3:**
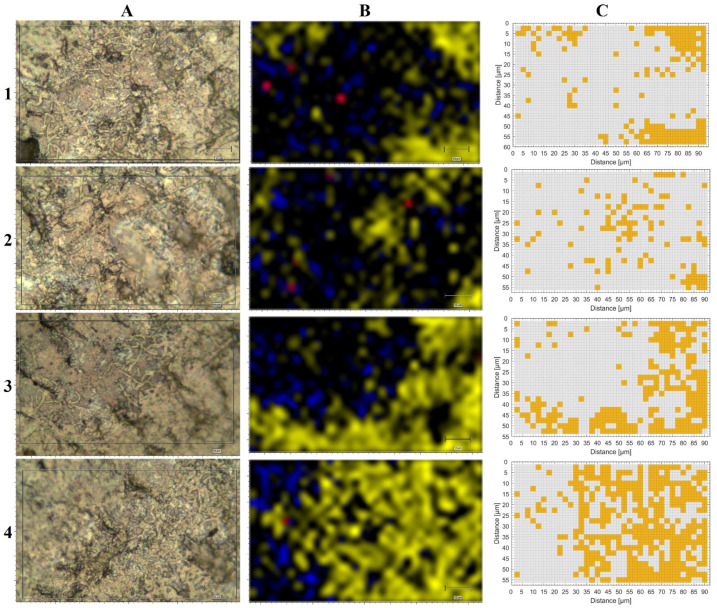
Randomly chosen areas of DSB on steel coupons (average area was approx. 5000 µm^2^ each). While **A(1–4)** represent microscopic images (grey rectangular frame shows measured area), **B(1–4)** show the corresponding Raman mappings. All spectra from **B(1–4)** were baseline-corrected via “intelligent fit” and normalized (z-score) by Wire 4.3 software before the Raman shifts were highlighted: 1524 cm^−1^ in yellow, 1448 cm^−1^ in blue, and 1015 cm^−1^ in red. The color scale starts from black at a normalized intensity of 1.5 and extends to the corresponding color (yellow, blue, and red) when the maximum intensity of the related Raman shift is reached. The color spaces were interpolated. **C(1–4)** show color coded measurement points (orange) that were predicted as *S. aureus* using the SVM model. For the prediction, the Raman spectra were treated with the appropriate pretreatment method required for the model (described in [18]).

Four different areas on the stainless-steel coupons were analyzed with an average of 798 Raman spectra per map. In the microscopical images of Figure 3A(1–4), the rod-shaped *Bacillus* cells could be observed, whilst staphylococci often formed clusters and frequently surrounded *Bacillus* rods. Highlighting Raman shifts for 1524 cm^−1^ in yellow, 1448 cm^−1^ in blue, and 1015 cm^−1^ in red (Figure 3B(1–4)) emphasized that carotenoid signals (yellow) dominate the Raman maps, especially in Figure 3B(4). The red spots, particularly visible in Figure 3B(1,2), were highly specific for *Bacillus* spores. No spores could be detected in the area shown in Figure 3B(3). The blue areas highlight a band that occurred in all microorganisms (1448 cm^−1^) but was more pronounced in *B. licheniformis* than in *S. aureus*. The clusters of orange squares in Figure 3C(1–4) visualize spots that were predicted as *S. aureus* by SVM. Taken together, approximately 19% of the Raman map C(1) was identified as *S. aureus*-positive, 13% of C(2), 30% of C(3), and 45% of C(4) (Figure 3). Although data from *B. licheniformis* were included in the model, no positive predictions were obtained for this species. However, this could be explained by the fact that the prediction model was not specifically trained for this purpose and the *Bacillus licheniformis* spectra contain less distinct features such as *Staphylococcus aureus* compared to the species included in the model. Furthermore, the spectra acquired on the DSBs are of lower quality than the reference spectra of pure cultures.

On stainless-steel coupons, not only could staphylococci be distinguished from *Bacillus* cells, but *Bacillus* spores could also be detected. Without a doubt, differentiating between vegetative cells and their spores might not only generally lead to a deeper understanding of complex DSB, but could also be helpful for biological hazard assessments. In healthcare settings, the spore former *Clostridioides difficile* is responsible for HAI and is of particular interest. The positive predictions for *S. aureus* with an independent model containing 21 species [18] demonstrate the great potential of such rapid analytical methods as Raman microscopy. Nevertheless, carotenoids must be considered a rather ideal distinguishing feature for Staphylococci and other pigmented species [19], which could have facilitated overall identification. The fact that *B. licheniformis* was not correctly predicted in the dual-species DSBs on stainless steel might support the assumption that Raman identification does not work that well for non-pigmented microorganisms and emphasizes the importance of reference data in predictive models. Figure 2 clearly shows that the spectra of *B. licheniformis* in monospecies DSBs differ from those obtained from a pure culture. Different studies have shown that various factors may impact the Raman spectra of microorganisms, such as the culture medium used [24,25] or the incubation time [26]. To obtain more accurate predictions for various sample types, the mathematical models would need to be fed with additional data, initially from monospecies biofilms. By extending or adapting the data set, Raman spectroscopic analysis techniques of DSB on metallic surfaces could thus become potentially promising screening methods.

To ensure more accurate, spatially resolved characterizations of DSB via Raman microscopy, reducing the laser focus diameter must be considered beneficial, because the larger the laser diameter, the more problematic mixture spectra become, as they contain superimposed information about different species and components [18]. Mathematically deconvolving these spectra in a meaningful way to obtain information on individual components might require either large amounts of data or may be limited to very clearly defined sample types. Likewise, three-dimensional distributions of contained species are an important factor especially when it comes to possible quantification. Within the applied experimental setup, it cannot be excluded that, for example, *Bacillus* cells are hidden under *S. aureus* cells. With a high confocality and a small laser diameter, three-dimensional predictions about biofilms could be made by measuring not only in X and Y directions but also in different Z distances. Liu et al. recently showed that a wide variety of chemical components such as proteins, bacterial cells, or glycolipids could be detected in the different biofilm matrices of *Pseudomonas* spp. and their corresponding three-dimensional spatial arrangement could be visualized and quantified [27]. Overall, the results are expected to show that the so-called matrix factorization Raman mapping method used could be a suitable tool to complement the conventional approaches to identify and visualize the chemical components in biofilm matrices [27].

Our study showed that Raman spectroscopy can be successfully used to detect DSBs, some individual species within DSBs, and spatial distribution. Our data, perhaps not surprisingly, highlighted a number of issues including the detection of non-pigmented bacterial species. This is the first step in investigating the role of Raman spectroscopy in the detection of DSBs and overall, our results are very encouraging. Yet, the biggest challenge will be to engineer the principle of Raman spectroscopy for a device that can be used in situ.

## Data Availability

Not applicable.

## Supplementary Materials

The following supporting information can be downloaded at: https://www.mdpi.com/article/10.3390/microorganisms10071369/s1, Supplementary Data S1. Exemplary microscopic images for Raman microscopy examined single cells of *Bacillus licheniformis* biofilms on steel coupons. Supplementary Data S2. MATLAB-Code for baseline subtraction and smoothing via low pass filter (LPF). Supplementary Data S3. Untreated and treated Raman spectra of data shown in Figure 1 (Raman mappings of *S. aureus* DSB on PVC).
