# Supplementary material for: Raman Microscopic Analysis of Dry-Surface Biofilms on Clinically Relevant Materials
Source: Microorganisms. 2022 Jul 7;10(7):1369. doi: 10.3390/microorganisms10071369 (PMC9319561; doi:10.3390/microorganisms10071369)
Supplement: Supplementary file 1 [file microorganisms-10-01369-s001.zip › Supplementary material Date S1.pdf]

Supplementary material

Date S1: Exemplary microscopic images for Raman microscopy examined single cells of *Bacillus licheniformis* biofilms on steel coupons. For better visibility of the respective cell, the crosshair was shifted slightly to the left in each case.

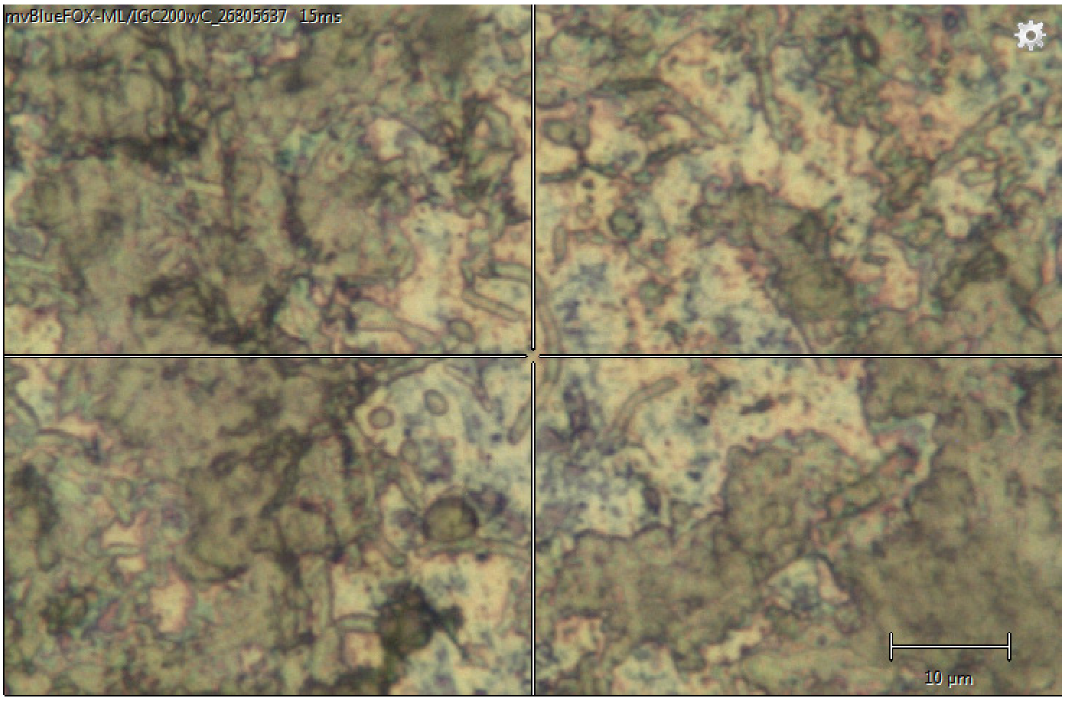

Targeted rod-shaped single cell of *Bacillus licheniformis*.

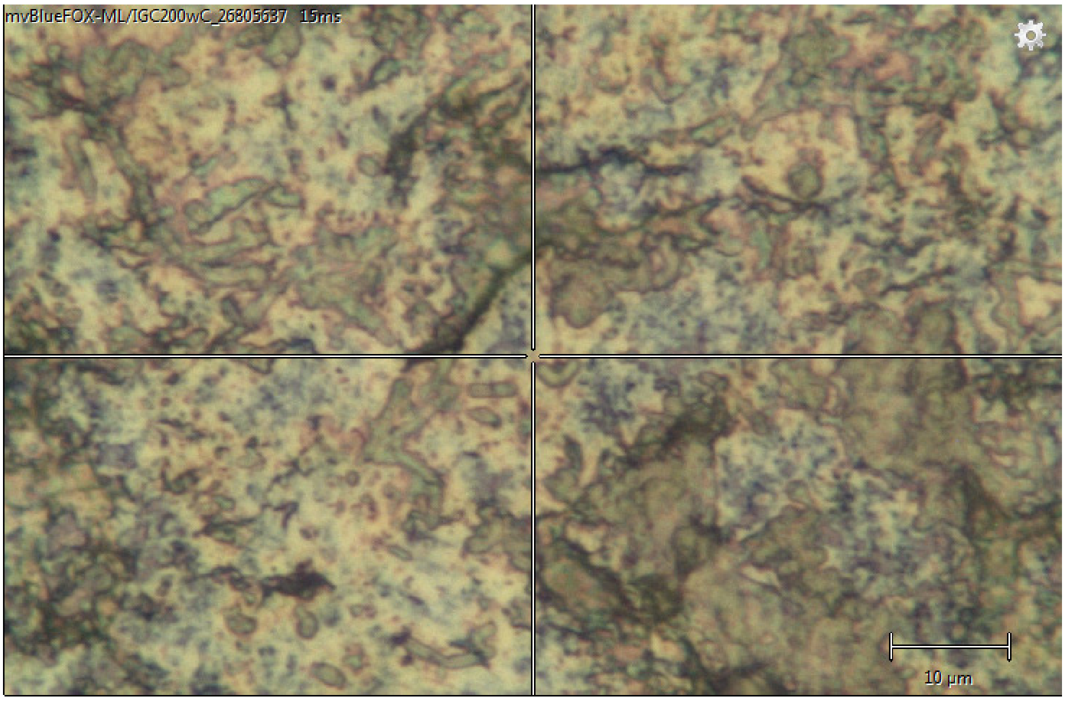

Rod-shaped single cell of *Bacillus licheniformis*.

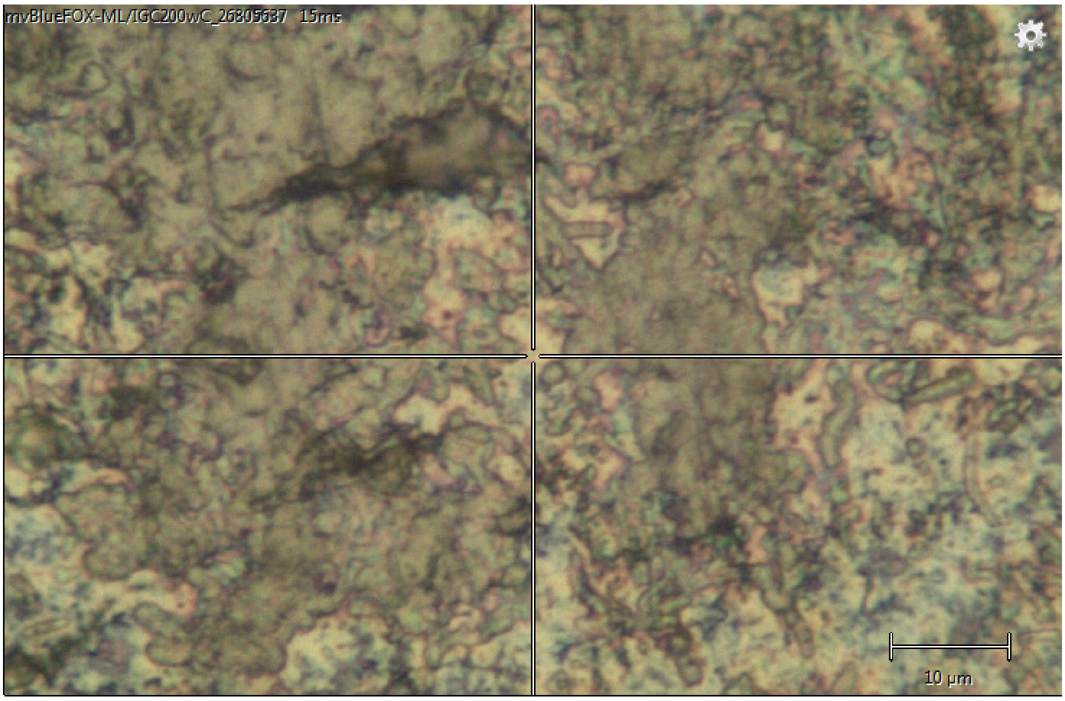

Targeted *Bacillus licheniformis* spore.

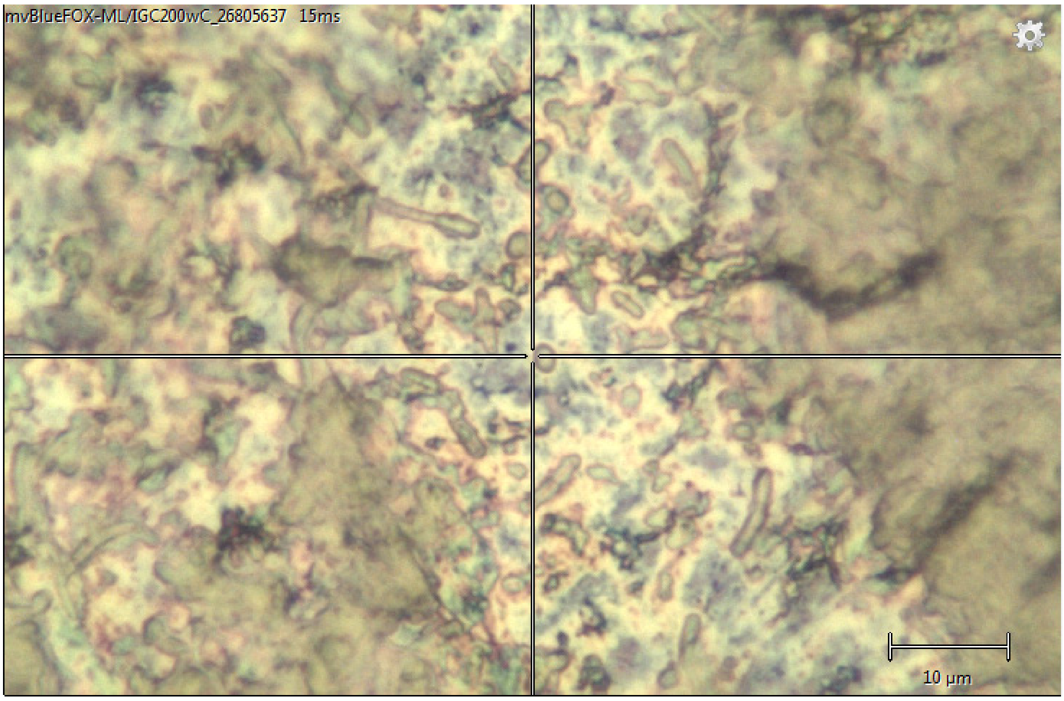

Targeted *Bacillus licheniformis* spore.
