# Supplementary material for: Raman Microscopic Analysis of Dry-Surface Biofilms on Clinically Relevant Materials
Source: Microorganisms. 2022 Jul 7;10(7):1369. doi: 10.3390/microorganisms10071369 (PMC9319561; doi:10.3390/microorganisms10071369)
Supplement: Supplementary file 1 [file microorganisms-10-01369-s001.zip › Supplementary material Date S2.pdf]

Supplementary material

Data S2: MATLAB-Code for baseline subtraction and smoothing via low pass filter (LPF).

```
%%Program code for LPF
%
function [Ampli]  = lpf(x, y, FT)
%
Delay = x;
Ampli = y;
%
d = length(x);
%
s1 = 0;
%
for i = d:-1:1
    s1 = (s1*(FT-1)+Ampli(i))/FT;
end
%
for i = 1:d
    s1 = (s1*(FT-1)+Ampli(i))/FT;
    Ampli(i) = s1;
end
%
for i = d:-1:1
    s1 = (s1*(FT-1)+Ampli(i))/FT;
    Ampli(i) = s1;
end

%%LPF application command
 %spectra=input data
 %dim = number of spectra
 %f = Raman-shift axis
for i = 1:1*dim
spectra_treat(i,:) = spectra(i,:) - lpf(f, spectra(i,:), 20) ;
spectra_treat(i,:) = lpf(f, spectra_treat(i,:),3) ;
end
%%Normalization via z-score
N = normalize(transpose(spectra_treat))
```
