# Supplementary material for: Raman Microscopic Analysis of Dry-Surface Biofilms on Clinically Relevant Materials
Source: Microorganisms. 2022 Jul 7;10(7):1369. doi: 10.3390/microorganisms10071369 (PMC9319561; doi:10.3390/microorganisms10071369)
Supplement: Supplementary file 1 [file microorganisms-10-01369-s001.zip › Supplementary material Date S3.pdf]

Supplementary material

Data S3: Untreated and treated Raman spectra of data shown in Figure 1 (Raman mappings of *S. aureus* DSB on PVC).

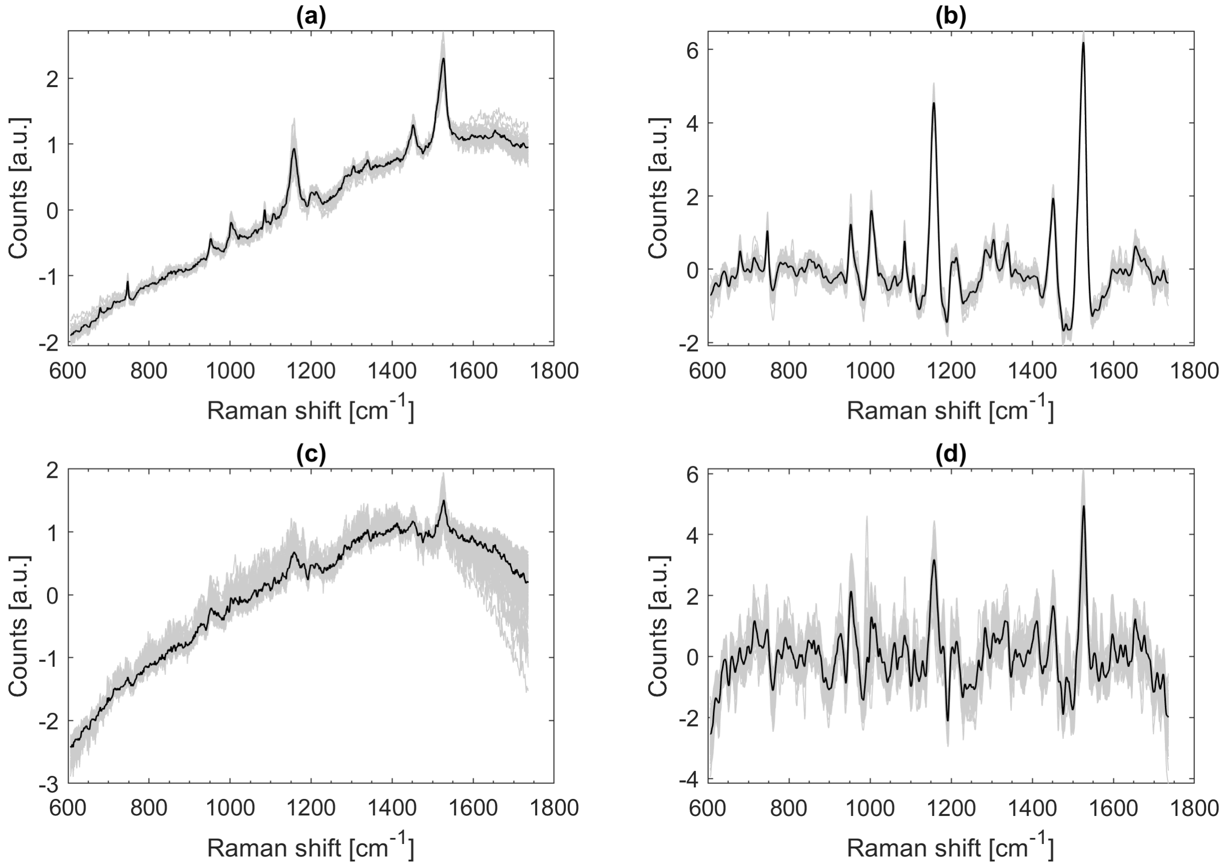

Untreated but normalized data of the smaller Raman map (1400 µm) recorded on PVC (**a**) and the same spectra treated (**b**). Untreated but normalized data of the larger Raman map (4000 µm) recorded on PVC (**c**) and the same spectra treated (**d**). The spectra of (**c**) show a stronger fluorescence background than the spectra of (**a**).
